# Effect of a Multi-Dimensional Intervention Programme on the Motivation of Physical Education Students

**DOI:** 10.1371/journal.pone.0085275

**Published:** 2014-01-15

**Authors:** Diana Amado, Fernando Del Villar, Francisco Miguel Leo, David Sánchez-Oliva, Pedro Antonio Sánchez-Miguel, Tomás García-Calvo

**Affiliations:** Faculty of Sport Sciences, University of Extremadura, Cáceres, Spain; Iran University of Medical Sciences, Islamic Republic of Iran

## Abstract

This research study purports to verify the effect produced on the motivation of physical education students of a multi-dimensional programme in dance teaching sessions. This programme incorporates the application of teaching skills directed towards supporting the needs of autonomy, competence and relatedness. A quasi-experimental design was carried out with two natural groups of 4^th^ year Secondary Education students - control and experimental -, delivering 12 dance teaching sessions. A prior training programme was carried out with the teacher in the experimental group to support these needs. An initial and final measurement was taken in both groups and the results revealed that the students from the experimental group showed an increase of the perception of autonomy and, in general, of the level of self-determination towards the curricular content of corporal expression focused on dance in physical education. To this end, we highlight the programme's usefulness in increasing the students' motivation towards this content, which is so complicated for teachers of this area to develop.

## Introduction

In the education field, amotivation towards learning is a topic of great concern that teachers have to contend with [16], [19]. In physical education, in particular, amotivation appears to a greater extent in some of the curricular contents, as is the case of dance (belonging to the corporal expression content block). This is because this content has traditionally remained in a second plane within this subject area, due to the lack of initial training of the physical education staff in dance teaching and consequently the lack of teachers' knowledge of the methodology to be used [34], [37]. This has led to proposals that are not very adequate for the students' learning needs [21]. Likewise, the students' amotivation may be due to social-cultural reasons, such as the largely female attribution to this type of content [17], [32].

In this sense, within the teaching-learning context, the motivation that drives learning and behaviour is studied, based on the personal factors of the surrounding environment [7], so students tend to engage with what they learn depending on specific objectives and on whether they perceive the experience as positive [49]. Thus, the type of motivation that has been associated with commitment to the area of physical education is intrinsic motivation [23].

This concept derives from the Theory of Self-Determination [14], which considers that behaviour is voluntary or self-determined, and distinguishes a continuum of motivation from the most self-determined to the least self-determined behaviour. It includes three types of motivation. Firstly, the most self-determined level is intrinsic motivation, which involves the students' engagement with the learning activities due to inherent reasons, such as the pleasure that the practice causes. To this end, it is usually associated with interest in the activity, with effort, with fun and with the sense of competence [12]. Then, extrinsic motivation appears, where the individual engages with the activity due to the external rewards it can offer, more than due to the pleasure he or she feels when carrying it out.

This motivation encompasses four types of regulation that evolve from a greater to a lesser degree of self-determination, respectively: integrated regulation (when the individual includes a behaviour within his or her main objectives and values (typical of the adult stage), identified regulation (when the individual grants value to an activity because he or she considers it important for him or herself), introjected regulation (when the individual has previously interiorised the external source of motivation, but still has not accepted the behaviour), and finally, external regulation (when the individual's behaviour is controlled by external sources such as rewards, threats and punishments). The continuum concludes with amotivation or the lowest level of self-determination, which refers to the absence of intrinsic and extrinsic motivation, representing a total lack of voluntarism or self-determination [14].

The research studies, developed around this theory, assume that although motivation as a variable is individual, it can be affected by social factors, such as the teacher's teaching style [47]. This has an impact on the student's motivation and engagement via the satisfaction of three basic psychological needs, namely, the need for autonomy, when the student considers him or herself to be the origin of his/her actions, the need for competence, when the student considers him or herself to be effective in all the interactions that occur, and the need for relatedness, when the student feels safe and connected to the others [12]. Thus, several studies suggest the use of a multi-dimensional teaching style that includes the teacher's support to autonomy, to competence and to relatedness as social factors than can be manipulated in the environment and that satisfies these three needs of the students [9], [38], [43], [45].

In this sense, the support to autonomy implies that the person in a position of authority, who, in this case, would be the teacher, allows the students to take decisions and be guided by their preferences [13]. To this end, strategies are used such as the democratic leadership style to foster students' active participation, making them feel important within the group, trying not to control or put too much pressure on the students to reduce competitiveness, making it possible for them to choose some activities, specifying the rules, explaining the objective of the activity so that there is greater engagement, using rewards related to the practice per se and placing emphasis on the students' social skills, providing them with efficient communication methods and assertiveness among them.

Support to competence refers to the extent to which a social context is structured, predictive and consistent, which will further permit satisfying the competence need [40]. In this case, physical education classes provide this social context where a series of techniques can be used to increase the perception of competence, such as adapting the exercises to the students' level, for them all to be able to be victorious. Activities can also be designed where success is evaluated through intrapersonal instead of interpersonal indicators, in order to foster effort and personal improvement, providing positive feedback so that students feel more secure and more aware of the triumphs they obtain. Furthermore, o can be provided as well as feedback related to the process and not to the result, in order to increase the skill perceived by the students, proposing different and accessible short, medium and long-term objectives that adapt to the students' needs.

Finally, the aim of support to relatedness is for people to feel they belong and are well-related within the social environment that surrounds them, because they receive affection, care and satisfaction [40]. In the area of physical education, a series of strategies can be used to favour the need for relatedness, such as setting aside some moments of the session for students to be able to establish personal relations and deal with topics that are unrelated to the actual session. Other strategies involve forming heterogeneous groups to boost the integration of all students, educating them in social skills so they learn to show empathy towards all their companions and towards the teacher; or programming cooperative exercises in order to favour taking mutual and dynamic group decisions to provoke the exchange of opinions, establishing strong and long-lasting relations.

Continuing in this vein, more and more research studies aim at increasing students' motivation during the physical education classes, through training programmes with teachers that have a bearing on didactic strategies to support the basic psychological needs. Initially, these studies focused more on fostering support to the students' autonomy during classes [6], [15], [39], [44], as previous studies showed a positive relationship between granting autonomy and motivating students during physical education classes [29], [30], [41], [43].

However, other authors [45] have chosen to develop a multi-dimensional intervention that covers support to the three needs, in order to verify the effect of an intervention programme based on the teacher's support to autonomy, competence and relatedness, on the motivation and satisfaction of the students' psychological needs. This research study was carried out over 7 sessions with three novice physical education teachers and their 185 students, aged between 14 and 18, from six secondary education school classes in the north-east of France. The results evidenced that the change in teaching style of the teachers led to a greater satisfaction of the students' psychological needs, more self-determined motivation and greater commitment in class.

Our study aims to carry out a more in-depth study of the application of multi-dimensional programmes to improve students' motivation in physical education. Its application with curricular corporal expression content focused on dance in secondary education is novel, as it is very important to study how these contents are being treated from the very first stages, because it is in this context where a larger number of people can be reached, seeking to increase their motivation, create adherence and for more people to practice it in the future [4], [18], [36]. In this regard, dance teaching in the real context of the physical education subject may arouse more feelings and emotions linked to movement and expressiveness in students, unlike normal physical education practices, which mainly seek motor competence [46].

Therefore, the objective of this research study is to verify the effect produced by a multi-dimensional programme on the motivation of physical education students during dance teaching sessions. This programme incorporates the application of teaching skills directed towards supporting the needs for autonomy, competence and relatedness. Based on this objective, the hypothesis established is for the application of the multi-dimensional intervention programme to produce an increase in the satisfaction of basic psychological needs and of self-determined motivation of students towards dance, in school physical education.

## Methods

### Ethics statement

Insofar as ethical rules are concerned, the study previously received the approval of the Ethics Committee of the University of Extremadura. All participants were treated in agreement with the ethical guidelines of the American Psychological Association with respect to consent, confidentiality and anonymity of the answers. Moreover, there was obtained an informed written consent from the parents and the headteachers of the school centres on the behalf of the minors/children participants involved in the study.

### Participants

A novice physical education teacher, with training in classical and contemporary dance, took part in the study, together with her 47 female (*n* = 29) and male (*n* = 18) students from two Secondary Education school classes in the Autonomous Community of Extremadura (Spain), aged between 14 and 16 (*M* = 14.84 years, *DT* = .48). All the students were in the 4^th^ year of secondary education and were selected according to the criterion of belonging to each one of the 2 classes they were grouped into. The teacher had one class with 27 students who were assigned as a control group and another class with 20 students who were assigned as an experimental group. The percentage of eliminated sample was higher than 5%, with a participation rate of 92.2% and with 4 invalidated questionnaires (7.8%) out of a total of 51 collected.

### Instruments

#### Basic psychological needs

To measure the satisfaction of basic psychological needs, the version of the Basic Psychological Needs Measurement Scale [48] translated into Spanish [26] was used. It was adapted by modifying the wording of the initial sentence, transferring it to the content of dance and corporal expression. Thus the instrument was preceded by the heading: “In dance and corporal expression classes in physical education…”, followed by 12 items (four per factor), which measure the satisfaction of autonomy (e.g.: The way the exercises are carried out coincide perfectly with the way in which I want to do them), satisfaction of competence (e.g.: I feel that I have progressed greatly with respect to the final objective that I had set out for myself), and the satisfaction of relatedness (I feel very comfortable when I carry out the exercises with the other companions). The internal consistency of the instrument was adequate both in the pre-test and in the post-test (pre-/post autonomy: .84/.80, pre-/post competence: .72/.70, pre-/post relatedness: .70/.75).

#### Level of self-determination

To evaluate the type of motivation of the students, the Questionnaire on Motivation in Dance and Corporal Expression [2] was used. This tool is headed by the statement: “I participate in dance and corporal expression classes in physical education…” followed by 20 items grouped into 5 factors that measure intrinsic motivation (four items, e.g.: “Because they are fun”), identified regulation (four items, e.g.: “Because I can learn skills that I could use in other areas of my life”), introjected regulation (four items, e.g.: “Because it is what I must do to feel good”), external regulation (four items, e.g.: “Because it is well looked upon by the teacher and companions”) and amotivation “four items, e.g.: But, I do not understand why we have to have these contents in Physical Education”). The reliability analysis reflected adequate internal consistency of this instrument at two moments of measurement (pre/post intrinsic motivation: .89/.81; pre/post identified regulation: .79/.81; pre/post introjected regulation: .70/.71; external regulation: .75/.76; pre/post amotivation: .72/.74).

The answers to both instruments were carried out on a Likert type scale with an answer range from 1 to 5, where 1 corresponded to *strongly disagree* and 5 corresponded to *strongly agree* with the formulation of the question.

### Procedure

Before carrying out the research study, all involved were informed about the process that they were going to follow, placing emphasis on the fact that participation was voluntary and that the data would be dealt with in a confidential manner, obtaining informed consent from the directors of the centres and the students' parents.

A quasi-experimental design was used, with two natural groups already established by a school centre, so it was not possible to respect randomisation. These two groups were divided into control and experimental. A multi-dimensional intervention programme was developed with the experimental group to increase the motivation of physical education students during the dance class. A dance teaching programme was developed with the control group, without the complement of the multi-dimensional programme to improve motivation.

The research was performed throughout the first and second terms at a secondary education school, with two weekly 50-minute sessions distributed throughout the week, which took place in the gymnasium of the centre. In order to cancel the effect on the teacher of learning the multi-dimensional programme to improve motivation, the 12 dance teaching sessions were initially developed (first term) with the control group, when the teacher was free to deliver them in accordance with her methodology. Thus, she did not receive any guidelines for the sessions. Later on, the teacher was taught how to master the multi-dimensional programme for its application during the second term and for another 12 sessions with the experimental group. These sessions were supervised at all times by the principal investigator in order to satisfy the objectives foreseen. To this end, a training programme was carried out with the physical education teacher.

The teaching programme lasted for 20 hours and was developed prior to the onset of the application of the intervention programme with the experimental group. The teacher attended a seminar on the theory of self-determination lasting for 6 hours, given by specialists, who explained in detail the basics of this model. Likewise, the teacher was instructed in the knowledge and application of the didactic strategies that she had to apply in the sessions, to support the needs for competence, autonomy and relatedness of her students, using, to this end, an adaptation of the strategies proposed by Amado [1].

To collect the research data, two measurements were carried out, pre-and post. The pre-measurement was carried out during the first session of each group, before this session was given, and the post-measurement was taken at the end of the process in both groups. Data collection consisted in administering a questionnaire to the students which they completed in the classroom without the presence of the teacher and in a climate that enabled them to concentrate without any type of distraction for 20 minutes. The principal investigator was present at all times to explain any doubts and make sure that the process was strictly followed.

### Data analysis

The SPSS 18.0 software program was used to process the data. Different tests were performed to verify the nature of the data, including the K-S test for independent samples to observe the normality of the group, the Rachas test for randomness and Levene's test for homocedasticity or equality between variances. The nature of the data was verified to be parametric, so parametric tests were chosen to be applied during the data analysis.

Thus, the data analysis strategies consisted of two parts: a preliminary analysis and a main analysis to observe the intervention effects. A multivariate analysis of variance (MANOVA) was carried out first with the pre-test data collected, to examine if there were statistically significant differences in the motivational variables between the two groups prior to the intervention and, therefore, to check if the two groups were homogenous or heterogeneous. Then, the main analysis was performed to discover the intervention effects in two senses. On the one hand, to verify the intra-group differences between the pre-test and post-test data collection, a *t* test was performed for related samples with each one of the groups. On the other hand, to analyse the inter-group differences between the pre-test and the post-test, a two-factor repeated-measures analysis of variance was performed (group: control and experimental)×2 (time: pre-test and post-test).

## Results

### Preliminary analysis

As a starting point, a MANOVA was performed of the dependent variables (satisfaction of the basic psychological needs and motivational regulation) with the pre-test data collected to analyse if the two groups were homogenous. In this sense, the results of this analysis reflected the existence of statistically significant differences at multivariate level (*F* (3, 44) = 4.25, *p* = .00; *η2* = .47). Subsequent ANOVAS reflected statistically significant differences in the satisfaction of competence (*F* (1, 45) = 8.88, *p* = .00; *η2* = .16) where the experimental group reflected higher scores than the control group, and in the satisfaction of autonomy (*F* (1, 45) = 6.82, *p* = .01; *η2* = .13), where the control group presented higher values than the experimental group.

### Effects of the intervention

On the other hand, to analyse the effects of the intervention in each group, a *t* test was firstly performed for related samples. The data analysis reflected a significant increase in the experimental group after carrying out the intervention of the perception of autonomy (*t* = −2.07, *p* = .05) and, in general an increase in intrinsic motivation (*t* = −1.06, *p* = .30), of identified regulation (*t* = −1.33, *p* = .20) and of introjected regulation (*t* = −1.34, *p* = .20), although these last three values did not reach significance. By contrast, a significant decrease in the perception of autonomy (*t* = 2.89, *p* = .00) and of relatedness (*t* = 2.61, *p* = .01) took place in the control group, as well as a significant increase in identified regulation (*t* = −2.19, *p* = .04). A marked, but not significant, increase of the need for competence was also reflected (*t* = −1.79, *p* = .08).

Later, taking into account the research design used and the differences in scores of the pre-test among groups, an ANCOVA (analysis of covariance) was used with the aim to separate the effect of the intervention program to the differences in the participants' selection. Therefore, with the aim to test the intervention program efficacy, an analysis of variance on post-test including participants' scores in pretest as covariate was conducted (Table 1).

**Table 1:** ANCOVA of the results measurements of the intervention program (post-test) for experimental and control group.

	Post-test				Contrast between groups	
	Experimental group		Control group			
Measure	*M*	*SD*	*M*	*SD*	*F*	*η2*
Intrinsic motivation	3.73	1.01	3.34	.95	1.76	.04
Identified regulation	3.53	.98	2.96	.92	3.82	.08
Introjected regulation	3.26	.96	3.06	.94	.28	.00
External regulation	3.18	.84	3.29	.94	.08	.00
Amotivation	2.19	1.22	2.63	1.24	1.58	.03
Competence	3.34	.79	3.33	.83	.01	.00
Autonomy	3.55	.69	2.95	.89	4.48*	.09
Relatedness	3.86	.78	3.50	.89	1.36	.03

*p*<.05*.

Although positive values were found in the experimental group comparing control group respecting intrinsic motivation, identified and introjected regulation, as well as satisfaction of needs of autonomy and relatedness, only a statistically significant effect was found in autonomy, being low the effect size (partial eta square (*η2* = .09)).

Regarding the results found in autonomy, after control “autonomy pre-test” (covariate) levels, a significant effect of the intervention on “autonomy post-test” (*F* (1, 45) = 4.48, *p* = .04, *η2* = .09) was found. That is to say, participants who received the intervention (experimental group), significantly increased their perception of autonomy at the end of the program comparing with control group which did not receive the intervention. However, no significant differences were found in the need for competence (*F* (1, 45) = .01, *p* = .93, *η2* = .00) or in the need for relatedness (*F* (1, 45) = 1.36, *p* = .25, *η2* = .03).

With regards to motivational regulation, no significant differences were found in the intrinsic motivation (*F* (1, 45) = 1.76, *p* = .19, *η2* = .04), in the identified regulation (*F* (1, 45) = 3.82, *p* = .06, *η2* = .08) or in the introjected regulation (*F* (1, 45) = .28, *p* = .60, *η2* = .00) but, if the level of significance of the identified regulation is observed, we can see that this is close to .05, showing an increase in this variable in the experimental group with respect to the control group.

## Discussion and Conclusions

Based on the postulates of the Theory of Self determination, the objective of this research was to verify the effect produced on the motivation of physical education students, of a multi-dimensional programme during dance teaching sessions, which incorporates the application of teaching skills aimed at supporting the needs for autonomy, competence and relatedness. The hypothesis that guided this work suggested that the application of the intervention programme would give rise to an increase in satisfaction of the basic psychological needs and of self-determined motivation of the students towards dance classes in school physical education.

After analysing the results, these showed that the students from the experimental group showed significantly greater satisfaction of the need for autonomy after applying the programme. In other words, the students who received a multi-dimensional programme based on the application of teaching skills aimed at supporting the basic psychological needs, underwent an increase in their perception of autonomy with respect to the control group that underwent a significant decrease. Thus, as the results point out and as several authors have also indicated previously, training physical education teachers in didactic skill learning to increase the autonomy of their students, represents a key element of best practices in physical education [3], [8], [9], [10], [27], [42].

With respect to the need for relatedness, an increase occurred in the satisfaction of this need in the experimental group with respect to the results found in the control group but, in this case, the values were not significant. Thus, they only show a slight positive trend, which must be reviewed in subsequent research studies. This trend may indicate that students from the experimental group begin to notice an increase in their sense of affiliation because, during the sessions, they identify a positive change in the teacher, which fosters more situations in which they have to interact with their colleagues, and they feel as if they are important elements within the group [35]. However, as indicated by other authors [45], for this increase to become significant, perhaps they need to notice these changes more or feel more linked to the group and increase relatedness with their class mates, or perhaps the time the programme lasted was not sufficient to appreciate changes in this variable.

The smaller values found in the control group could have been caused because the teacher, on not having received any guidelines to develop the dance teaching sessions with this group, gave these sessions using her own methodology, which corresponded to a more traditional style that focused on reproducing a model. This is something that has traditionally been done insofar as these contents are concerned: “Traditionally, dance has been geared towards repeating and memorising movement patterns, focusing on reproducing, memorising or copying” [33, p. 219]. This type of methodology means that students focus their attention on executing an established model and place all their efforts on carrying out all the movements correctly, so their relationship with the rest of their companions may be reduced, to exchange their opinions and actively participate in the teaching-learning process [20].

With respect to competence, on conclusion of the intervention programme, no significant changes took place in the satisfaction of this need, between the experimental group and the control group. In this vein, some authors [45] conducted research with similar characteristics, using different sports and physical education and at the end of the intervention programme they pointed out the lack of significant changes in the perception of competence. They also indicated that to notice a substantial change when starting to learn new tasks, time is necessary, and perhaps the duration of the programme (six 50-minute sessions) was not sufficient for the students to feel more competent. In this regard, in this study, the duration of the intervention programme was probably insufficient, too, as when greater autonomy is granted to the students (as occurred in the experimental group) when learning novel contents such as dance, the perception of skill is reduced. Thus, more practice time could be necessary to improve the specific skill and their perception of competence [22], [24], [25], [28].

With respect to the motivational regulations, it is worth pointing out that an increase occurred in intrinsic motivation, identified and introjected regulations in the experimental group with respect to the results found in the control group, but the values were not significant. Maybe, this values were not significant because it must be taken into account that in the school context, students normally feel very controlled because attendance is compulsory, they do not find the contents and learning activities fun and their work is constantly assessed [5], [21]. However, the increase that has occurred in the experimental group in intrinsic motivation is important because it indicates that there has been an increase in the satisfaction and pleasure encountered by the students during the dance lessons within the contents delivered in physical education [12]. In this sense, the increase in identified and introjected regulations may be conditioned by the actual characteristics of this area, which make students consider the curricular content of corporal expression focused on dance as important for them (identified regulation), or feel guilty if they do not carry out the activities (introjected regulation) due to reasons that are external to them, but not due to the interest that the practice arouses in them [11].

In this line, some previous works have recognised a positive and significant relationship between the teacher's behaviour on providing support to autonomy, and the students' self-determined motivation during the physical education classes [29], [30], [41], [43]. However, it must be pointed out that these studies were crosscutting and therefore only one measurement was carried out. On the contrary, on analysing the studies that include one measurement at the start of the programme or pre-test and another at the end of it or post- test (as occurs in this work), we find that, in agreement with the results found in this study, other authors [31], [45] also pointed out that the support provided by the physical education teacher to the basic psychological needs did not produce a significant increase in intrinsic motivation and identified regulation.

After conducting this research, the main conclusion drawn is the usefulness of a multi-dimensional intervention programme applied during the curricular content of corporal expression focused on dance and physical education, which incorporates teaching skills aimed at supporting the basic psychological needs, in order to significantly increase the students' perception of autonomy and, in general, their level of self-determination. Also to be taken into account is the fact that these variables are related to behavioural, cognitive and more positive affective consequences, placing special importance on the adherence to the practice of physical activity, a priority objective of physical education classes.

Despite this, it must be pointed out that in this study, the control and experimental groups used were not very numerous and therefore, the results obtained must be classified as preliminary ones. To support these results, future work should increase the sample and use more experienced teachers, as the teaching intervention programme has been carried out with a novice teacher who was very interested in participating and learning new teaching strategies. But, this type of programme requires a lot of time and effort and the degree of commitment of more experienced teachers in this type of training must be verified, and, above all, to this type of content that usually is left in a second plane within the contents delivered in physical education.

Another aspect of interest to be dealt with in the future, would be to increase the duration of the intervention programme and use a longitudinal design with various measurements at different moments in time, to observe the evolution and changes recorded in the variables analysed, with the passing of time, and to examine if the trends that arise based on the development of this research, could become significant in time. Thus it would be possible to obtained more generalisable conclusions. Likewise, it would be appropriate to complete this study with qualitative data based on the students' experiences and impressions, which would favour a more in-depth analysis of the emotional changes that they may experience during the sessions with this type of physical educational content, and later be able to place emphasis on positive aspects and try to avoid, insofar as possible, factors that may cause more negative feelings.
